# Ayurveda education: A student's perspective

**DOI:** 10.4103/0974-7788.64410

**Published:** 2010

**Authors:** Namyata Y. Pathak

**Affiliations:** *Department of Kayachikitsa, Smt. K. G. Mittal Ayurveda Hospital, Mumbai, India*

"India has or rather *had* the knowledge of the spirit, but she neglected matter and suffers for it. The West has the knowledge of matter but rejected the spirit and suffers badly for it. An integral education, which could, with some variations be adapted to all the nations of the world, must bring back the legitimate authority of the spirit over a matter. Yet, knowledge of the matter must also be fully developed and utilized …" (The Mother at Sri Aurobindo Ashram)

## INITIATION INTO AYURVEDA

A fascination for the physiology of water and ionic exchanges in the nephron curiously drew me to medicine, quite conveniently brushing aside 2 years of preparation for the Indian Institute of Technology entrance examination. After being a guinea pig for different systems of medicine as an asthmatic patient for many years, I had finally found a peaceful night's rest during the monsoons, with Ayurvedic treatment consisting of decoctions of *pathyadi* and *dashmoola* with *kankayani vati*. From a crippling dependence on salbutamol inhalers to a rather rounded facial appearance due to a florid Cushing's syndrome developed by an adulterated corticosteroid administration by a quack practitioner, I had suffered enough. Wanting to be an empowered patient rather than a guinea pig and following many dinnertime conversations with my mother, who felt a need to safeguard Ayurveda; and my chartered accountant father, whose love for nature and medicine continues to inspire me, I took the plunge into the unknown world of Ayurveda at the age of 18. Unsurprisingly, the choice seemed irrational to everyone else, as it involved turning down a conventional medicine seat at Grant Medical College to start my Ayurvedic education at R. A. Podar Ayurvedic Medical College. I was frequently mocked at; overtly then, covertly now. My choice was emboldened by conversations with Makrand Dave a mystic poet who saw no reason why scientific disciplines could not bridge across both ancient and modern medicine.[1] By creatively and scientifically correlating ancient insights and modern discoveries, he had earlier encouraged other physicists, physicians and scientists to pursue that path and that too, quite successfully.

## MY FIRST FORAYS INTO AYURVEDA

While my initial dream involved becoming an empathetic Ayurvedic physician, over time it has morphed, often and unpredictably, to now make room for becoming a physician-scientist. This path has involved transient forays into medical journalism, pediatrics, tropical medicine and geriatrics. Along the way, my interests have crystallized into studying the role of Ayurveda in the treatment of geriatric disorders like Parkinson's disease. I share a snapshot of the past 8 years as a student — through an alteration of preconceived notions, a challenging of my comfort zones, the learning that resurrected child-like joy, mentors and exposures that have nudged me to a growing appreciation of the uncertainty of human knowledge. Five decades hence, it may fulfill the pursuit to become a liberal, empathetic Ayurvedic physician-scientist.

## WITH AN EMINENT VAIDYA: STRENGTHS OF AN APPRENTICESHIP

At her clinic brimming with patients, Vd. Amee Parikh, a gold medalist from Gujarat Ayurveda University, Jamnagar, lovingly and openly taught me Ayurveda as she had painstakingly learnt from the masters. She taught me what she successfully practiced over 25 years. Concurrently with the undergraduate training, I experienced patient responses to Ayurvedic personalized medicine, the art of pulse diagnosis and the strength of intuition at her clinic. It was much later that I realized how rare her openness to teach was; in contrast to traditional practice, where there was a fierce instinct to guard skills and knowledge. Until now this conservationist attitude has probably helped to preserve tradition along centuries, but today it seems to stem from a lurking insecurity of competition and lack of credit for the tradition's contribution. Yet the few students who choose to pursue Ayurveda practice after graduation feel a need to be trained under these live *vaidya* traditions. The Government of India has formalized this for selected graduate students under the *guru-shishya parampara* at the Rashtriya Ayurveda Vidyapeeth, New Delhi.

However, one wonders if apprenticeship with *vaidya* traditions could be given a distinct status and brought closer to main-line academia at undergraduation itself.[2,3] This would clearly be a win-win situation for the traditions, academia, students and even research as newer approaches could be guided, potential leads could be generated and research in fundamentals could be cultivated. Dynamism of clinical application and a wide range of contextual interpretations are in fact essential features of a *sutra* that are brought out by meditation. Observing stalwarts like Vd. C. P. Shukla, who contemplate and clinically apply the sutras, students can witness, at firsthand, the current understanding of ancient insights. Yet genuine reverence is not compatible with blind acceptance. There can be a renaissance in Ayurveda if we were open to the revision of *shlokas* and expansion of existing *sutras* based on new knowledge in life sciences. Such adaptability was a norm in Ayurveda until a few centuries ago. Ayurveda's eternal nature was never meant to result in stagnation.

## INSTITUTIONAL TRAINING: DISAPPEARANCE AND REAPPEARANCE OF QUESTIONS

Dr. D. N. Shukla, our *Sharira Rachana* teacher in the first year, made special efforts to help me understand Ayurvedic medical philosophy in Sanskrit. Meanwhile, institutional learning got us acquainted with the 'whats' and some 'hows' of the classical texts, developed basic diagnostic skills and demonstrated some possible prescription patterns. The curriculum proposed by Central Council of Indian Medicine also incorporates some aspects of modern medical sciences, offering a conglomeration of medical information under the pretext of 'integrative' knowledge.[4] On shaky grounds, we students had to take an initiative for extra-institutional training even for the practical approaches of modern medicine. Though I was a curious kid in school, that trait withered in the first year of graduation study . Answers seemed vague, were based on different fundamentals which could not be questioned or debated until one knew enough. Five years later, these questions re-sprouted when patients sought my advice. I wondered whether I was a channel of propagating a belief or truth. What would be an adequate dose for the effect targeted? When should we treat? What did the patient really expect at that disease stage? When should we refer? An intra- and inter-system mode of referring patients was almost absent in my reasonably varied experience. The attitudes of practitioners in Ayurveda are not very open, nor are those of our conventional medicine brethren. Suddenly, there were alarm bells. Did our education train us to believe that we knew it all? Shouldn't it aim to make us aware about what we do not know, and allow us to cultivate a tentativeness when we are in doubt? We misused the flexibility of Ayurveda to win arguments, became intolerant of questions and launched defensive attacks when criticized. It was incomprehensible that all events could be understood in a singular model. Can't there be several models to represent reality? Wasn't the purpose of education to make us aware of the limits of our knowledge and how to push the boundaries of ignorance? Can we be more complete by the complementarity of other scientific knowledge?

## ATTITUDINAL SHIFT: EXCITEMENT OF OPPORTUNITY

Frustrated, I found my next mentor, Dr. Ashok Vaidya, a clinical pharmacologist whose contributions to Ayurveda and modern medicine spanning four decades reflect his true reverence and dedication to both systems of healing. Viewed as a convert to Ayurveda by many conventional doctors and a critic by some *vaidyas*, he nudged me to unlearn my methods of learning. With his multi-disciplinary team at Medical Research Center, Kasturba Health Society, he enhanced my trans-system approach to diverse domains of knowledge. At times, it was overwhelming to get exposed to malaria, *pragnyapradha*, neurosciences, medicinal plants, diabetes on one hand with history of medicine, international health issues, Hindu philosophy and information technology on the other. Probably to become an effective physician-scientist, one needs to be a well-rounded generalist.[5] I learnt how modern sciences that shaped modern medicine could contribute to, and gain from, Ayurveda. For example, I had experienced an acute cervical pain syndrome that had developed as a result of suppression of an explosive sneeze. Suppression of 13 types of natural reflexes, *vegadharana*, is a unique concept in Ayurveda for the causation of various diseases, and Ayurveda has a description for their specific management. We surprisingly discovered other case reports of complications due to suppressed sneezing. Wouldn't it be interesting to explore *vegadharana* in the etiopathogenesis of other diseases, like the contribution of social suppression of flatus to the formation of intestinal diverticulosis?[6] We could build new bridges across diverse epistemologies of Ayurveda and modern medicine, as reverse pharmacology has done on paths of drug discovery.[7] Yet again, a respite from the didactic educational system rekindled the earlier vision, while exciting ideas and dreams shaped.

## THE POSTGRADUATE YEARS: EDUCATION AND RESEARCH

Though there is a quantitative extension of exposure to academic institutions, qualitatively, the paucity of the nature of clinical material and inadequacy in training of clinical skills continue. Patwardhan *et al*. have recently demonstrated these serious flaws in a large nationwide survey of undergraduates, postgraduates and teachers of Ayurvedic medical colleges. They show that students at the end of training remain ill-equipped to handle both the simple emergencies of primary health care and the specialized Ayurvedic procedures like *panchakarma*.[8] Unfortunately, theoretical debates without bedside relevance further intensify frustration of students during this period of postgraduation. Access to latest scientific literature is nearly absent in our libraries, where the potential of library and information science lies untapped. Without a foundation of knowledge acquisition, processing and retrieval systems, students in this system feel obsolete outside their academic institutions.

For my postgraduate (PG) dissertation, I had naively hypothesized the possibility of 'neuro-regeneration' in patients of Parkinson's disease (PD). I have painfully realized the inadequacies to design a study for PD that demands (1) specialized clinic with attendance of adequate number of patients, (2) clinical acumen for diagnosis and (3) research methodology with understanding of sample size. To traverse these difficulties, I had to contact neurologists at public hospitals and private practice. Sensing an enthusiasm, the busy residents and faculty at the Department of Neurology, KEM Hospital, Mumbai, kindly took an initiative to teach me. Through a rapport built with few senior neurologists in India with a wide experience in PD, I got an opportunity to address patients of the support groups of PD and Movement Disorder Society. Gaining confidence after an interdisciplinary exposure, it became feasible to conduct a successful camp to study multiple aspects of the disease. Meanwhile, after a brief exposure in the neuroscience laboratory of Dr. Vidita Vaidya at Tata Institute of Fundamental Research, Mumbai, which works on neurogenesis in the adult brain, the complexity and depth of neuronal plasticity research became evident.[9] The diversity and sophistication of concepts and methods involved in neuro-regenerative studies at different levels of biological organization, from molecules to man, were mind-boggling. I then realized that the lack of meticulous study designs influenced the outcome and relevance of PG dissertations. Merely applying randomized controlled trial designs and probability-based biostatistics to Ayurveda is less likely to enhance research in disease management. We must cognize the *pramanas*, the Ayurvedic modes of evidence,[10] the availability of other trial designs and develop robust skills of observational therapeutics and experiential documentation. As a student, I wonder if this learning, which was achieved through self-motivation and initiative and tremendous support from family, teachers and friends could be facilitated as a norm by models like "Bilateral Education."[11]

## IN CONCLUSION: THE LARGER PICTURE

As the proposed Health Council of India is actively reconsidering the revision of the curricula, there is hope that the best elements from Western education and Ayurvedic tradition will be synergized in the interest of patients. This may involve elements of translational research across systems of medicine for evidence-based practice. Current educational systems — both conventional and Ayurvedic —should develop humane and confident doctors with a patient-centric integrity who imbibe attributes ascribed to an ideal *guru, shishya* and *vaidya* as described by Charaka.[12] Such physicians would be aware of their functional knowledge, acknowledge intra- and inter-system strengths and weaknesses and unstintingly adapt to the rapid advances in the practice of medicine. Ayurveda stands to contribute to areas of preventive and promotive health care, disease risk management, early-stage management and prevention of complications of chronic diseases, and disease-modifying treatments. As future practitioners, we expect to be so trained as to enhance local, national and international health care through integrative Ayurveda equipped with these strengths, with dignity and a high level of self-esteem.

## EDITORIAL NOTE

"Ayurveda Education: A Student's Perspective" written by a fresh postgraduate from Ayurveda brings to fore the students perspective of education in Ayurveda. It is not a scientific paper (although as per journal policy it has undergone peer review), rather, it is an essay supported by some relevent observations and references. It is hoped that a healthy discussion/debate may ensue on the issues besetting education in Ayurveda.

Dr. Urmila Thatte, Chief Editor.

## References

[1] Dave M, Vaidya R, Raut A (2006). Yuvati Purani quotation: Bhartiya Vidya Bhavan's – Swami Prakashanand Ayurvedic Research Centre: A Journey of twenty years.

[2] Singh RH, Nanal V, Bhatt N (2007). Plenary session on educational reforms: Conclave Proceedings: Transforming traditions for tomorrow's health Ayurvedic Education.

[3] Brooks MA (2009). Medical education: Tyranny of competency. Perspect Biol Med.

[4] Department of Indian Systems of Medicines and Homoeopathy. Government of India Annual Report 1999–2000.

[5] Grossman DC, Michael B, Valtin H (1999). Great issues for medicine in the twenty-first century: Ethical and social issues arising out of advances in the biomedical sciences. Annals of New York Academy of Sciences.

[6] Pathak N, Raut A, Vaidya A (2008). Acute cervical pain syndrome resulting from suppressed sneezing.J Assoc Physicians India.

[7] Vaidya AD (2006). Reverse pharmacology correlates of Ayurvedic drug actions. Indian J Pharmacol.

[8] Patwardhan K, Gehlot S, Singh G, Rathore HC The Ayurveda Education in India. How well are the graduates exposed to basic clinical skills? Evid Based Complement Alternat Med 2009 in press.

[9] Ming GL, Song H (2005). Adult neurogenesis in the mammalian central nervous system. Annu Rev Neurosci.

[10] Pathak N, Vaidya AB, Kothari M, Doshi J (2009). Pathogenesis of amla-pitta due to high salt intake. Current discoveries confirm Charaka.

[11] Stumpf SH, Shapiro SJ (2006). Bilateral integrative medicine, obviously. Evid Based Complement Alternat Med.

[12] Charak Samhita Charak Samhita. Vimana Sthana.

